# Syncope following Pfizer BioNTech (bnt162b2) vaccination unmasking Brugada syndrome

**DOI:** 10.1016/j.heliyon.2023.e18716

**Published:** 2023-07-26

**Authors:** Mohammad Altermanini, Mhd Baraa Habib, Dania Alkhiyami, Khaled Ali, Mohamed Salah Abdelghani, Tahseen Hamamyh, Ahmad Elyas, Mawahib Elhassan

**Affiliations:** a Department of Internal Medicine, Hamad Medical Corporation, Doha, Qatar; b Hamad Medical Corporation, Doha, Qatar; c Community Medicine Department, Hamad Medical Corporation, Doha, Qatar; d Department of Cardiology, Heart Hospital, Hamad Medical Corporation, Doha, Qatar

**Keywords:** Brugada, Coronavirus, COVID-19, Vaccine, BNT162b2, Syncope

## Abstract

The Brugada syndrome is an uncommon inherited condition associated with increased risk of ventricular tachyarrhythmias and sudden cardiac death. Different triggers including fever are well known to precipitate the Brugada pattern on electrocardiogram. We report a patient who presents with syncope, two days after the first dose of the BNT162b2 vaccine due to fever-related unmasking of Brugada syndrome.

## 1 Introduction

Brugada syndrome was initially discovered in the 1990s, and firstly characterized with unexplained sudden cardiac death [1]. Brugada syndrome had characteristic electrocardiographic abnormalities in right precordial electrocardiogram (ECG) leads that predispose to malignant ventricular tachyarrhythmias leading to eventually sudden cardiac death. Patients with Brugada syndrome are often asymptomatic and unaware of their hereditary susceptibility [2]. Therefore, the diagnosis of Brugada syndrome is usually made after arrhythmic syncope or sudden cardiac arrest [3]. The ECG signs of Brugada syndrome are often unnoticed but can be unmasked mainly by fever and/or medications causing potent sodium channel blockade that provokes arrhythmia [4]. In this case, we report a case of syncope unmasking Brugada syndrome, which was provoked by fever after receiving BNT162b2 vaccine, in a previously healthy patient.

## 2 Case presentation

44 years old Indian gentleman without significant medical history presented after an episode of syncope. The history was taken from a friend who witnessed the syncope episode at work. The patient suddenly lost consciousness then developed generalized tonic clonic (GTC) seizure with tongue bite which lasted for around 10 minutes. The patient had post-ictal tiredness with no bladder or bowel incontinence. There was no history of any identifiable precipitants. The patient regained consciousness after 1–2 minutes. He reported a daylong of subjective fever, which he attributed to the coronavirus disease-2019 (COVID-19) vaccine. He received the first dose of the BNT162b2 vaccine 48 hours before the presentation. He denied recent travel or any sick contacts. The patient had a similar episode of syncope approximately 3 years ago, in which he had a brief syncopal attack lasted for few seconds and he didn't seek any medical advice and did not receive any medications; the patient denied any family history of sudden cardiac death.

**Fig. 1:**
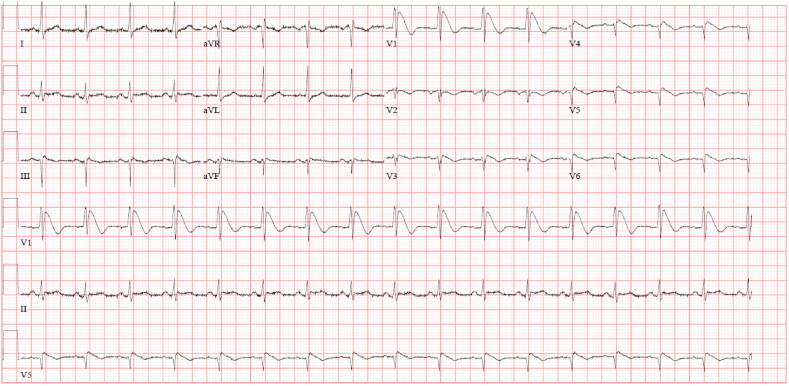
ECG upon admission shows sinus rhythm with Coved STsegment elevation >2mm in V1–V3 followed by a negative T wave.

In the emergency department, the patient was afebrile, alert and fully conscious, with stable vital signs so no medications were administered. Blood tests and CT head were ordered in view of new seizure. Suddenly, he collapsed and had ventricular tachycardia followed by ventricular fibrillation which required cardiopulmonary resuscitation (CPR) and defibrillation for 3 minutes. The patient denied having any chest pain at any stage during his presentation. After the CPR, a 12 lead ECG showed normal sinus rhythm with Brugada pattern in V1–V2, with S1Q3T3 with minimal ST elevation in the inferior leads (Fig. 1). His lab tests including cardiac enzymes are shown in Table 1. CT Pulmonary Angiogram was done, and it excluded pulmonary embolism. CT Coronary angiogram showed minimal stenosis in the left anterior descending artery (LAD). Cardiac magnetic resonance imaging (MRI) was done and consecutively a subcutaneous defibrillator was implanted successfully. The patient was stable and he was discharged from ICU.

**Table 1:** Lab tests in the emergency department.

Test	Value w/Units	Normal Range
WBC	7.9 × 10^3/uL	4.0–10.0
RBC	5.7 × 10^6/uL	4.5–5.5
Hgb	12.3 gm/dL	13.0–17.0
Platelet	180 × 10^3/uL	150–400
Urea	3.6 mmol/L	2.5–7.8
Creatinine	75 μmol/L	62–106
Sodium	141 mmol/L	133–146
Potassium	4.1 mmol/L	3.5–5.3
Chloride	110 mmol/L	95–108
Bicarbonate	21 mmol/L	22–29
Magnesium	0.98 mmol/L	0.70–1.00
Bilirubin T	6 μmol/L	0–21
Total Protein	59 gm/L	60–80
Albumin Lvl	31 gm/L	35–50
Alk Phos	62 U/L	40–129
ALT	63 U/L	0–41
AST	39 U/L	0–40
**Troponin-T HS**	**257 ng/L**	**3**–**15**

**Fig. 2:**
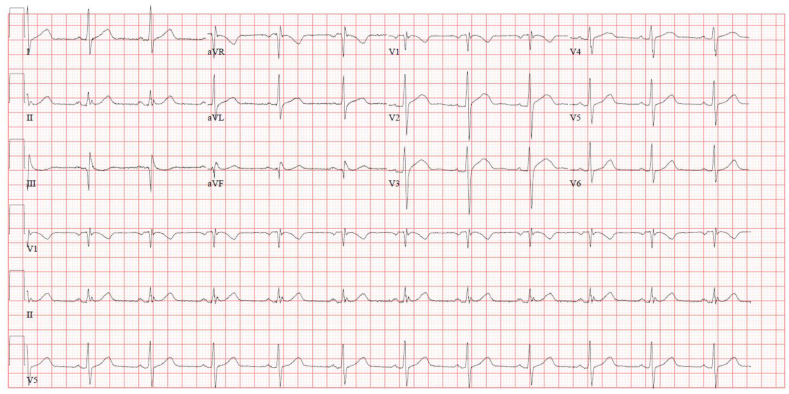
Repeated ECG during the follow-up visit showing sinus rhythm with Q3 and inverted T wave on V1.

Follow-up visit after 3 months, the patient was in good condition with no more attacks. Vital signs were normal. ECG was repeated and showed sinus rhythm with Q3 and T wave inversion on V1 (Fig. 2). No genetic testing was done.

Written consent was obtained prior to the writing of this report.

## 3 Discussion

The COVID-19 outbreak has been announced as a pandemic by the World Health Organization (WHO) in 2020 [5]. COVID-19 manifests mainly as a respiratory syndrome that includes pneumonia and can progress to acute respiratory distress syndrome [6]. However, acute cardiovascular events including arrhythmia were reported with COVID-19 infection particularly in intensive care unit (ICU) settings and may be contributing to disease outcomes [7]. The mechanism of cardiac arrhythmias in COVID-19 infection is multifactorial and could be contributed to hypoxia, myocarditis or drugs side effects [8]. On the other hand, some cardiac arrhythmias can be due to inherited syndromes such as Brugada syndrome (BrS), long QT syndrome (LQTS), short QT syndrome (SQTS), and catecholaminergic polymorphic ventricular tachycardia (CPVT) which are triggered by COVID-19 infection [9]. Fever was reported as a precipitating factor for around 18% of the cardiac arrests in patients with symptomatic Brugada syndrome [10]. Chang et al. reported a male patient presented with symptomatic Brugada syndrome triggered by Covid 19-infection-induced fever [2].

Since the beginning of COVID-19 pandemic, several effective vaccines were developed for SARS-CoV-2, yet there are still many questions regarding their safety. One of the most common reported side effects was fever, and it was mainly observed after the second dose of BNT162b2 mRNA Covid-19 Vaccine within the first one to two days after vaccination [11]. A recent study reported that the incidence of cardiac arrhythmias as a side effect of BNT162b2 vaccine was the lowest among all COVID-19 vaccines [12].

Considering the acute history, the short interval between the administration of vaccination and the presentation of the patient with cardiac arrest, and the exclusion of other common causes; BNT162b2 vaccine-induced fever which unmasked Brugada syndrome is the most likely precipitant factor in our patient.

A similar case was reported by Okawa et a, suggested that ECG screening is necessary before the initial COVID-19 vaccination to find any asymptomatic unknown Brugada syndrome, to reduce the risk of sudden cardiac arrest in such patients [13]. Our case is among the very few cases demonstrating the presence of Brugada syndrome after receiving the NT162b2 vaccine, yet we still lacking the privilege of having a pre-vaccination ECG with an obvious Brugada pattern.

## 4 Conclusion

We would like to highlight the importance of performing additional precautions and preventive measures like ECG monitoring and antipyretic treatment with Covid 19 vaccination, as it may reduce the cardiac arrhythmias that might be developed due to fever, in patients with underlying conduction disorders such as Brugada syndrome.

## Author contribution statement

All authors listed have significantly contributed to the investigation, development and writing of this article.

## Data availability statement

Data will be made available on request.

## Additional information

No additional information is available for this paper.

## Funding

This research did not receive any specific grant from funding agencies in the public, commercial, or not-for-profit sectors.

## Declaration of competing interest

The authors declare that they have no known competing financial interests or personal relationships that could have appeared to influence the work reported in this paper.
